# High Incidence of Multiple Viral Infections Identified in Upper Respiratory Tract Infected Children under Three Years of Age in Shanghai, China

**DOI:** 10.1371/journal.pone.0044568

**Published:** 2012-09-07

**Authors:** Guocui Zhang, Yunwen Hu, Hongping Wang, Lu Zhang, Yixi Bao, Xiaoming Zhou

**Affiliations:** 1 Shanghai Public Health Clinical Center, Affiliated to Fudan University, Shanghai, China; 2 Department of Clinical Laboratory, Second Affiliated Hospital of Chongqing Medical University, Chongqing, China; University of Liverpool, United Kingdom

## Abstract

**Background:**

Upper respiratory tract infection (URTI) is a major reason for hospitalization in childhood. More than 80% of URTIs are viral. Etiological diagnosis of URTIs is important to make correct clinical decisions on treatment methods. However, data for viral spectrum of URTIs are very limited in Shanghai children.

**Methods:**

Nasopharyngeal swabs were collected from a group of 164 children aged below 3 years who were hospitalized due to acute respiratory infection from May 2009 to July 2010 in Shanghai. A VRDAL multiplex PCR for 10 common respiratory viruses was performed on collected specimens compared with the Seeplex® RV15 ACE Detection kit for 15 respiratory viruses.

**Results:**

Viruses were detected in 84 (51.2%) patients by VRDAL multiplex PCR, and 8 (4.9%) of cases were mixed infections. Using the Seeplex® RV15 ACE Detection kit, viruses were detected in 129 (78.7%) patients, 49 (29.9%) were co-infected cases. Identified viruses included 37 of human rhinovirus (22.6% of cases), 32 of influenza A virus (19.5%), 30 of parainfluenzavirus-2 (18.3%), 23 of parainfluenzavirus-3 (14.0%), 15 of human enterovirus (9.1%), 14 each of parainfluenzavirus-1, respiratory syncytial virus B and adenovirus (8.5%), 8 of coronavirus 229E/NL63 (4.9%), 6 of human bocavirus (3.7%), 5 each of influenza B virus and respiratory syncytial virus A (3.0%), 3 of parainfluenzavirus-4 (1.8%), 2 of coronavirus OC43/HKU1 (1.2%), and 1 human metapneumovirus (0.6%).

**Conclusions:**

A high frequency of respiratory infections (78.7%) and co-infections (29.9%) was detected in children with acute respiratory infection symptoms in Shanghai. The Seeplex® RV15 ACE detection method was found to be a more reliable high throughput tool than VRDAL method to simultaneously detect multiple respiratory viruses.

## Introduction

Upper respiratory tract infections (URTIs) are common in children. They can occur three to eight times per year, or even more in very young children [1], [2]. These respiratory infections maybe caused by a wide range of microorganisms. Approximately 80% of cases are viral [3]. Some common viruses responsible for respiratory infections include Respiratory Syncytial Virus (RSV), human Rhinovirus (hRV), human Enterovirus (hEV), Influenza A Virus (IAV), Influenza B Virus (IBV), human Metapneumovirus (hMPV), Coronavirus (CoV), Parainfluenza Virus (PIV), Adenovirus (AdV), and human Bocavirus (hBoV) [4]–[9]. The similar clinical presentations of patients infected by various respiratory viruses make etiological diagnoses difficult when doctors simply make decisions based only on physical symptoms [10]–[12]. This can lead to the misuse of drugs, i.e. oseltamivir and amantadine are two effective drugs used to treat IAV infections but have no effect on URTIs caused by other respiratory viruses.

Rapid high throughput diagnostic methods can identify the causative pathogens at an early stage of the illness [2], [13]. The multiplex RT-PCR technique is currently the method of choice for the diagnosis of respiratory viruses due to its high sensitivity and specificity [5]. The application of multiplex design RT-PCR allows for the simultaneously detection of multiple infections. Co-infection of hMV and SARS-Cov (Severe Acute Respiratory Syndromes associated Coronavirus) had contributed to the severity of cases during last 2003 SARS worldwide epidemic [14], [15]. Thus, the possibility of co-infection must be considered before making clinical decisions.

There are limited data on the infection profiles of URTI cases in Shanghai children younger than 3 years of age due to the lack of appropriate diagnostic tools. This is one important reason for the over-prescription of unnecessary antibiotics to children presenting with fever in pediatric hospitals in Shanghai. With support from the Chinese Key SciTech Programme for Infection Control (under The Eleventh Five-year Framework), our laboratory began a pilot program to monitor virus pathogens responsible for URTIs symptoms in Shanghai. We adapted a VRDAL method for this surveillance pilot program based on a previously published paper by Australian scientists [16]. This multiplex RT-PCR method can detect 10 viruses at the same time, but cannot differentiate between hRV and hEV, PIV-1 from PIV-3, or RSV A from RSV B. The Seeplex® Respiratory Virus Detection assay (SeeGene Inc., Seoul, Korea) was introduced to the respiratory research community in 2007 and can efficiently differentiate 15 respiratory viruses [5]. Thus, the Seeplex® RV15 ACE Detection kit was also used to assess the virus profiles.

## Materials and Methods

### Patient Samples

Nasopharyngeal aspirate swabs were collected from 164 patients under 3 years of age (male: female = 1.28∶1) with fever and acute respiratory infection (ARI) symptoms, hospitalized at the Pediatric Inpatient Department of Xinhua Hospital from May 2009 to July 2010. Around 1000 of such patients were received medical services in this hospital sporadically distributed all over the year. We randomly select average of three patients from the hospital to take part in the respiratory virus surveillance program based on their hospital registration number per week. These patients had not received any other virus test service from this hospital. Their samples were taken before they had received any further clinical therapy. No outbreak event for respiratory disease linked these patients during the full research period.

Informed written consent was obtained from parents. The Internal Research Ethics Committee of Shanghai Public Health Clinic Center, FuDan University, granted ethics approval.

Each nasopharyngeal swab was mixed with 4 mL of viral transport medium (minimal essential medium with 2% fetal bovine serum, 100 U/mL penicillin, 100 mg/mL streptomycin, 20 mg/mL amphotericin B, 40 mg/mL neomycin). Then, 0.2 mL of each sample was used for analysis. The rest was frozen at −70°C.

### Extraction of Viral Nucleic Acids and Reverse Transcription

Viral nucleic acids were extracted from 200 µL of each sample using the High Pure Viral Nucleic Acid Kit (Roche Applied Science, Castle Hill, Australia) following the manufacturer’s instructions. Extracted nucleic acids were eluted in 35 µL RNase-free ddH_2_O and stored at −70°C.

Synthesis of cDNA was performed using RevertAid™ First Strand cDNA Synthesis kit (Fermentas International, Ontario, Canada). In brief, 8 µL extracted RNA was mixed with 1 µL random hexamer (0.2 µg/µL) and 3 µL RNase free ddH_2_O, and the mixture heated for 3 min at 80°C. Then, 4 µL 5× reaction buffer, 2 µL 10 mM dNTPs, 1 µL RNase inhibitor (20 U/µL), and 1 µL reverse transcriptase (200 U/µL) were added to the mixture in the same centrifugal tube, and the solution was incubated at 37°C for 90 min and at 94°C for 2 min. For negative controls, sterile distilled water was used instead of RNA extracted from real specimens. Synthesized cDNA was stored at −40°C until use.

### VRDAL Multiplex PCR

The VRDAL multiplex PCR, which was designed for simultaneously detecting 10 respiratory viruses, was performed as described previously [16]. Three groups of reactions were performed for each sample. Group 1 included reactions for IAV, IBV, and AdV. Group 2 included reactions for RSV A/B and picornaviridae (with primers specific for all enterovirus and rhinovirus). Group 3 included reactions for PIV1-3. Internal positive control of BVDV and negative control of nuclease-free water were included in each group of reactions. Primers used for first and second round PCR amplifications are referred in original paper [16]. Subtyping of hEV, hRV, and PIV depends on the sequencing results from acquired PCR products. For the first round amplification, 2.5 µL cDNA was added to a 22 µL master mixture containing 500 nmol first round primers (100 nmol for picornavirus), 25 mmol MgCl_2_, 2.0 mmol dNTPs, and 0.3 U Hotstar® Taq polymerase (Qiagen GmbH, Hilden, Germany). The PCR reaction protocol included a 15 min denaturation step at 94°C, then 35 cycles of 30 s at 94°C, 30 s annealing at 55°C, and 60 s extension at 72°C, then a final elongation step at 72°C for 7 min. For the second round amplification, 1 µL of each product from the first round was transferred to a freshly prepared master mixture containing primers for second round reactions. PCR conditions were the same as used in first round reactions. Final PCR products were separated by 30 min electrophoresis (10 V/cm) on a 2% agarose gel prestained with ethidium bromide.

Contamination of PCR products between tests was strictly avoided by following the standard PCR operation guideline.

### Seeplex® RV15 ACE Multiplex PCR

The Seeplex® RV15 ACE Detection kit (Seegene Inc., Seoul, South Korea) is designed for simultaneously detection of 15 respiratory viruses, including IAV, IBV, RSVA, RSVB, AdV, hMPV, PIV1-4, hRV, hEV, coronavirus 229E/NL63, and OC43/HKU1, and hBoV. Sample cDNAs were tested in a 3-tube reaction following the protocol supplied by manufacturer. Tube-1 contained primer set A targeting AdV, 229E/NL63, and PIV1-3. Tube-2 contained primer set B targeting OC43/HKU1, hRV, RSV A/B, and IAV. Tube-3 contained primer set C targeting hBoV, IBV, hMPV, PIV4, and hEV. Positive controls included a mixture of all 15 virus clones. The negative control contained only ddH_2_O. To set up these multiplex PCR reactions, 4 µL of each 5× RV15 multiplex primer sets (A or B or C), 3 µL 8-methoxypsoralen (8-MOP) solution, 10 µL 2× Multiplex Master Mix (Hotstart® Taq DNA polymerase and dNTPs included), and 3 µL cDNA template were mixed together to a final 20 µL volume. Reaction mixtures were first denatured at 94°C for 15 min, following by 40 cycles of denaturation at 94°C for 30 s, annealing at 60°C for 90 s, extension at 72°C for 1.5 min, and a final extension step at 72°C for 10 min. PCR products were visualized by electrophoresis on a 2% agarose gels and staining with ethidium bromide.

### Single PCR

For those co-infection cases identified by VRDAL or RV15 multiplex PCR, single PCRs were run for each genre of virus to reconfirm the co-infection status. For conflict results from VRDAL and RV15 tests, single PCRs were also run to dissolve controversies.

PCR run condition was the same as the condition used by Seeplex® RV15 ACE multiplex PCR except that only one tube contained one pair of PCR primers targeted to individual genre of virus was used for each reaction.

Primers targeted to 14 individual viruses for single PCR were listed in **TABLE 1**. Primers for hMPV was not listed here because only one hMPV case was detected in this paper without any controversy between two multiplex PCR tests.

**Table 1:** Primers for Single PCR.

Virus	Target	Forward primers	Reverse primers	Length
229E/NL63	Nucleocapsid	GGT ACT CCT AAG CCT TCT CG	TGC ACT AGG GTT AAT GAA GAG G	370 bp
OC43/HKU1	Nucleocapsid	AGG AAG GTC TGC TCC TAA TTC C	TGC AAA GAT GGG GAA CTG TGG G	450 bp
RSV-A	F	TGA CCC ATT AGT GTT CCC CTC TGA TGA AT	CTT CTG GCC TTR CAG TAT ARG AGC AGT	228 bp
RSV-B	N	AAG ATG CAA ATC ATA AAT TCA CAG GA	CAC TAT AAA GAT ACT TAA AGA TGC TGG ATA TCA	103 bp
IAV	M	CTT CTA ACC GAG GTC GAA ACG TA	GGT GAC AGG ATT GGT CTT GTC TTT A	155 bp
IBV	N	GTC CAT CAA GCT CCA GTT TT	TCT TCT TAC AGC TTG CTT GC	145 bp
PIV1	HN	CCG GTA ATT TCT CAT ACC TAT G	CTT TGG AGC GGA GTT GTT AAG	317 bp
PIV2	HN	CCA TTT ACC TAA GTG ATG GAA T	GCC CTG TTG TAT TTG GAA GAG A	204 bp
PIV3	HN	ACT CCC AAA GTT GAT GAA AGA T	TAA ATC TTG TTG TTG AGA TTG	103 bp
PIV4	P	CCT GGA GTC CCA TCA AAA GT	GCA TCT ATA CGA ACA CCT GCT	204 bp
hBoV	NP1	GAC CTC TGT AAG TAC TAT TAC	CTC TGT GTT GAC TGA ATA CAG	354 bp
hRV	5′NCR	GCA CTT CTG TTT CCC C	CGG ACA CCC AAA GTA G	380 bp
hEV	5′NCR	CCC CTG AAT GCG GCT AAT	CAA TTG TCA CCA TAA GCA GCC A	103 bp
AdV	Hexon	CCT ACG CAC GAT GTG ACC ACA GAC CG	GTG TTG TAG GCA GTG CCG GAG TAG GG	213 bp

### Definition of Seasons

Seasonal definition was defined by the Weather Bureau according to average air temperature over five continuous days. In 2009–2010, summer in Shanghai was defined from 6th May 2009 to 8th Oct 2009, fall from 9th Oct 2009 to 12th Nov 2009, winter from 13th Nov 2009 to 20th Mar 2010, and spring from 21st Mar 2010 to 9th June 2010.

### Statistical Analysis

Comparisons between two groups were evaluated by the Pearson’s *Chi*-square test or the Fisher’s exact test as appropriate for sample size.

## Results

### VRDAL Multiplex PCR

A total of 164 samples were tested by VRDAL multiplex PCR. Viruses detected by this test are summarized in **TABLE 2**. Since 4.9% of the samples were identified as co-infected, the overall number of positive samples is less than the total number of positive results for individual viruses. Overall, 84 positive samples (51.2%) and 90 positive results for individual viruses were detected by the VRDAL PCR method.

### Seeplex® RV15 Multiplex PCR

Expected products can be amplified by single round Seeplex® RV15 multiplex PCR. They are clearly distinguishable by agarose gel electrophoresis. Overall, 129 positive samples (78.7%) were detected from the 164 samples tested by the Seeplex® RV15 method. Among them, 26 samples showed double infections, 16 cases were triple infections, 6 cases were quadruple infections, and 1 case of quintuple infections was also detected. A total of 165 positive results for individual viruses were obtained and 29.9% of samples showed multiple infections. Identified viruses are summarized in **TABLE 2**.

### Single PCR

Total of 127 reactions were re-performed by single PCR for those co-infected cases. Twenty of them were negative. Other 84.3% (107/127) reactions showed agreed results to Seeplex® RV15 multiplex PCR. Most non-agreed tests were performed for PIV-3 (11/19) and PIV-2 (5/27). Their positive product bands were isolated from gel after electrophoresis separation, and were sequenced to confirm their typing by comparison with PIV genomes retrieved from GenBank (data not shown). We speculated that those non-agreed tests were resulted from the non-optimum selection of primers for single PCR due to the hypevariation of PIV-3 and PIV-2 genomes. Other non-agreed tests included each one for RSV-A, IBV, OC43/HKU1 and AdV.

**Table 2:** Positive sample detections by two multiplex PCR tests.

Virus Detected	Seeplex® RV15	VRDAL
	Positive number (%)	Positive number (%)
hRV	37 (22.6%)	42 (25.6%)^a^
IAV	32 (19.5%)	22 (13.4%)
PIV2	30 (18.3%)	10 (6.1%)^b^
PIV3	23 (14.0%)	b
hEV	15 (9.1%)	a
PIV1	14 (8.5%)	b
AdV	14 (8.5%)	10 (6.1%)
RSV B	14 (8.5%)	4 (2.4%)^c^
229E/NL63	8 (4.9%)	**–**
hBoV	6 (3.7%)	**–**
RSV A	5 (3.0%)	c
IBV	5 (3.0%)	4 (2.4%)
PIV4	3 (1.8%)	**–**
OC43/HKU1	2 (1.2%)	**–**
hMPV	1 (0.6%)	**–**
Total Positive	129 (78.7%)	84 (51.2%)

a the total number of hRV and hEV.

b the total number of PIV1-3.

c the total number of RSV A and RSV B.

“–”: not included tests. Multiple virus infections were detected in a single sample by both PCR tests.

### Subgroup Detection of Viruses by Seeplex® RV15 Multiplex PCR

**TABLE 3** summarizes positive detections among subgroups using the Seeplex® RV15 multiplex PCR. There was no difference in positive detection rate between male and female patients (75% *vs.* 83.3%, χ^2^ = 1.671, P = 0.196). The infection rate in the 13–24 month age group was significantly higher than in the 8–12 month or 25–36 month groups (92.6% *vs* 55.9% and 47.9%, χ^2^ = 26.614, P<0.001). Detection rates among three diagnosed diseases showed no statistically significant difference.

**Table 3:** Positive samples detected by Seeplex® multiplex PCR by subgroup.

Patients	Tested Samples	Positive	*P*-value
**Gender**			
Male	92	69	
Female	72	60	?^2^ = 1.671, P = 0.196
**Age**			
< = 7 months	3	3	not included in calculation
8–12 months	59	33	
13–24 months	54	50	
25–36 months	48	23	?^2^ = 26.614, P<0.001
**Diagnosis**			
Rotaenteritis	2	2	
Pneumonia	3	3	
URTIs	159	124	?^2^ = 1.399, P = 0.497

URTIs: Upper respiratory tract infections.

### Seasonal Virus Detection by Seeplex® RV15 Multiplex PCR

Detection rates of 5 viruses exhibited seasonal fluctuation as summarized in **TABLE 4**. PIV1-4 infections were common in samples taken in the spring (15/22), a rate significantly higher than in the other seasons, although more PIV1-4 infections were detected in summer samples (44/89). The highest rate of hEV infection occurred in summer, with 14 of the 15 total cases reported that year. Most IAV and IBV infections were detected in either summer (25) or winter (8), accounting for 33 of the 37 total cases that year. Infections with hRV were most common in autumn at 11 of all 24 samples, and in summer at 21 of all 89 samples. In winter, RSVA/RSVB infections were seen at 10 of 29 samples. None of the other detected viruses showed significant seasonal fluctuations.

**Table 4:** Seasonal detection of five genre of viruses.

Season	hEV		RSV A/B		hRV		IAV/IBV		PIV1–4	
	(+)	(-)	(+)	(-)	(+)	(-)	(+)	(-)	(+)	(-)
**Spring**	1	21	0	22	4	18	3	19	15	7
**Summer**	14	75	6	83	21	68	25	64	44	45
**Fall**	0	24	3	21	11	13	1	23	6	18
**Winter**	0	29	10	19	1	28	8	21	5	24
?^2^	9.477		16.031		13.999		8.080		18.473	
P	0.012		0.000		0.003		0.040		0.000	

“+”: numbers for positive samples, “−”: numbers for negative samples.

### Body Temperature Associated with Viruses Detected

All 164 patients tested had fever, including 29 cases of low fever (37.3–38.0°C), 103 cases of middle fever (38.1–39°C), and 32 cases of high fever (39.1–41.0°C). Virus detection rates stratified by fever level were 75.9%, 78.6% and 81.3% respectively. For all these groups, the first three most predominant viruses identified were the same: hRV, IAV and PIV2.

Apart from fever, 34 patients had just symptoms of cough, 15 cases had just runny nose, while 67 cases had cough accompanied by runny nose. The other 48 cases had no cough or runny nose symptoms. Virus detection rates of these four symptom groups were 91.2% (cough), 80.0% (runny nose), 85.1% (cough and runny nose), and 60.4% (no cough or runny nose), respectively. Distribution of detected viruses in these groups showed no significant difference (χ^2^ = 1.393, P = 0.499). The first three predominant viruses identified for these groups were the same as hRV, IAV, and PIV2.

### Co-infections Identified by Seeplex® Multiplex PCR

Co-infection rates grouped by individual respiratory viruses ranged from 83.3% for hBoV (5/6) to as low as 33.3% for hEV (5/15) (**TABLE 5** **)**.

**Table 5:** Co-infection rate by virus.

Virus	Co-infection cases	All detected	Rate
PIV 1–4	57	70	81.4%
hRV	20	37	54.1%
IAV/IBV	17	37	45.9%
AdV	10	14	71.4%
RSV A/B	8	19	42.1%
hBoV	5	6	83.3%
CoV	5	10	50.0%
hEV	5	15	33.3%

### A Brief Comparison of Seeplex® RV15 and VRDAL Multiplex PCR

The same 164 specimens were tested using both multiplex PCR methods. Altogether, 129 specimens (79.7%) were detected as positive by Seeplex® RV15 PCR, while only 84 specimens (51.2%) were detected as positive by VRDAL PCR. All VRDAL positive specimens were also found to be positive using Seeplex RV15 PCR, with the same detected viruses. Thirty-one specimens (18.9%) were both negative by Seeplex RV15 and VRDAL PCR detection kits.

**Figure 1:**
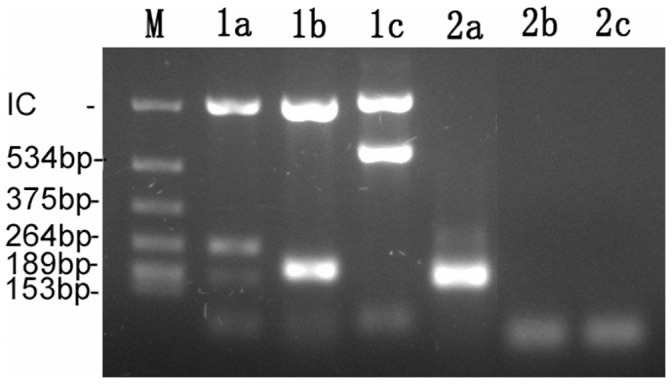
One example analyzed both by Seeplex® RV15 and VRDAL multiplex PCRs. lane “M”: molecular PCR marker, size of each bands were annotated in left; “IC”: 850 bp internal positive control, all presented in lane 1a–1c; “1”: one sample (A2-1319T) analyzed by Seeplex® RV15 method; lane “1a”, “1b”, “1c”: three individual reaction groups, showed a quartile infections included PIV2(264 bp, 1a), PIV3(189 bp, 1a), FluA(206 bp, 1b) and HboV(579 bp, 1c); “2”: same sample analyzed by VRDAL method; lane “2a”, “2b”, “2c”: three individual reaction groups, showed single FluA infection detected (196 bp, 2a) in lane 2a with negative results detected both in lane 2b and 2c.

Even when accounting for those viruses (coronavirus, hMPV, and hBoV) not included in VRDAL, the VRDAL method showed lower test sensitivity compared to Seeplex® RV15 PCR (**TABLE 2**). The VRDAL method indicated that 5.9% of cases had multiple infections, whereas Seeplex® RV15 PCR indicted that 29.9% had multiple infections. The overall concordance for these two methods was 58.9%. One example showed difference between Seeplex RV15 and VRDAL was demonstrated in **Fig. 1** **.**

## Discussion

Conventional viral culture and antigenic-antibody reactions are often time-consuming with low sensitivity for viral detection. They may take as long as one week to acquire test results. A number of studies aimed to develop and evaluate multiplex PCR for the detection of respiratory viruses [1], [4], [17], [18]. Multiplex PCRs often produce false positive bands due to cross dimers formed between PCR primers. Therefore, test of compatible primers is essential for developing a good multiplex PCR assay [5], [11]. In the past, difficulties in the design of compatible primers limited the detection range of multiplex PCR assays.

In this study, two multiplex PCRs were compared to identify viruses prevalent in Shanghai children suffering from ARIs. The Seeplex® RV15 assay showed higher sensitivity than the VRDAL multiplex PCR which was currently applied in some of diagnosis laboratories. It detected 27.5% more positive specimens than the VRDAL method (78.7% *vs.* 51.2%). In addition to those viruses not covered by VRDAL (PIV4, hBoV, CoV and hMPV), the Seeplex® RV15 assay showed 40% higher sensitivity for detection of IAV, PIV1-3, IAV, AdV, and RSV A/B. For example, Seeplex® RV15 yielded a 8.5% infection rate for AdV compared to 6.1% by VRDAL. Since VRDAL requires a two round amplification procedure, whilst Seeplex® RV15 is performed using only one round, the improvement in sensitivity is attributed to a better primer design strategy adopted by Seeplex® RV15 assay as dual-priming oligonucleotide system.

When conventional methods are used for detection of respiratory viruses in clinical specimens, 33.0–56.4% positive results are obtained, but multiple infections cannot be detected [5], [18]. A previous study reported that Seeplex® multiplex PCR obtained 56.0–75.2% positive results from clinical samples, with a co-infection rate of 6.0–8.0% [9]. In this study, we reported that 78.7% of URTI specimens were positive for respiratory viruses, and 29.9% revealed multiple viral infections. This high rate of respiratory virus co-infection has not been reported previously. It also cannot be revealed out by other tests like viral culture or immunoblot, due to the lack of high throughput capability.

Another respiratory virus monitoring study from Beijing reported a co-infection rate of 17.8% in 365 URTI children using multiplex PCR during Oct, 2006 to Apr, 2007. Among them, 75.38% of co-infection cases included RSV infection [19]. Shanghai reported only a 0.2% co-infection rate in 11,214 URTI children by direct immunofluorescence assay [20]. This difference in co-infections rate is likely caused by the lack of simultaneous detection capability of direct immunofluorescence assays.

An initial pathogenic virus inducing opportunistic infections by other seasonal epidemic viruses may cause this high rate of virus co-infections. Co-infections in winter were more likely to include RSV, while in spring, co-infections were more likely to include PIV. We propose that just one of the co-infected viruses is the real pathogen for URTIs. Most possibly, this pathogenic virus should be the virus other than the seasonal epidemic circulated viruses.

Overlapping infections by two co-infected viruses may also exert dual malignancy on infected patients. In the case of bronchiolitis, co-infection increased the number of patients demanding to be admitted to the intensive care unit from 15.2% to 34.1% (P<0.001) due to decreased health status [21]. In such cases, the goal of medical treatment should be the simultaneously control of all co-infected viruses.

In our study, PIV 1–4 and hBoV were found most frequently presented in co-infection cases at rates of 80.0% (56/70) and 83.3% (5/6). PIV 1–4 are viruses circulated seasonally in spring and summer, and thus appear unlikely to be the primary pathogen for URTIs. Most of hBoV presented with other viruses. We speculate that the development of hBoV pathogenicity may require its reciprocal interaction with other co-infecting viruses.

It is interesting to know the clinical impact of multiple respiratory infections in this study. However, due to the limitation of this surveillance program, we are currently lack the clinical information of those patients included in this work. We will keep keen to such themes in future.

Our study reports significantly different rates for single infection and co-infections from previous studies. Wang had reported that the most frequently identified respiratory viruses were hMPV (15.4%), IAV (13.2%), and RSV (8.8%) in Shanghai URTI children during the period from Oct, 2006 to Aug, 2007 [9]. Another study had reported that the three most predominant viruses were RSV A/B (24.7%), hBoV (24.5%), and hRV (15%) identified in Shanghai and NanXiang children during period from Oct, 2006 to Sep, 2008. In this study, the most prevalent respiratory viruses were identified as hRV (22.6%), IAV (19.5%) and PIV2 (18.3%). Specimens collected from outbreak events can greatly alter the infection profile for respiratory diseases. So caution was exercised to avoid inclusion such specimens in our tests. Thus, the different findings from the three reports cited are attributed to the yearly changes in Shanghai. The high incidence of RSV reported in Shanghai and NanXiang children was explained by the increased isolation rate of all viruses due to an extremely cold winter in 2007.

Another previous study reported that RSV A/B, PIV-3 and AdV were the 3 most common viruses, with isolation frequencies of 17.7%, 2.8% and 2.2%, respectively, identified in 11,214 Shanghai children with ARI during a four years period (2003–2006) [20]. However, they did not include hRV, hEV, hMPV, hBoV, and CoV in their analysis due to lack of a multiplex PCR technique at that time.

Few of our patients were infected with hMPV (1 case), hBoV (6 cases), CoV (10 cases), or AdV (14 cases); thus the seasonal distribution of these 4 viruses cannot be assessed in this study. For other viruses, in concordance with other studies, hEV was most frequently isolated in summer, while RSV A/B was most frequently observed in winter, hRV in fall, and PIV1–4 in springs. The IAV/IBV virus showed two peaks in seasonal distribution in Shanghai, a city with a temperate climate. This pattern was different from more northern cities like Beijing. Thus, more accurate forecasting of seasonal epidemic viruses could help healthcare professionals better prepare for combating respiratory infections in children. Since hRV and hEV showed different seasonal distribution patterns, Seeplex® multiplex PCR may be superior over the VRDAL PCR method, which can differentiate between hRV and hEV and so as to correctly detect seasonal changes in respiratory virus distribution.

Virus distribution did not differ between male and female patients. However, age groups <7 months and 13–24 months showed higher infection rates. For infants less than 7 months, this high rate may be due to the under development of their immune systems after birth. For children aged 13–24 months, increased rates may reflect the accumulation of infections. After 24 months, maturation of the immune system restrained a further increase in infections and decreased infection rates.

In conclusion, we showed here that the Seeplex® RV15 ACE detection method performed well than VRDAL method to detect multiple viruses in respiratory infection cases, due to its board inclusion of multiplex PCR primers designed for 15 respiratory viruses. In addition, an unusual high frequency of respiratory infections (78.7%) and co-infections (29.9%) was detected in children with acute respiratory infection symptoms under three years in Shanghai by using Seeplex® RV15 ACE detection method. Dominance and seasonal relevance of viruses were discussed. Interesting on the clinical impact of multiple virus infections were largely increased for future studies.
